# Risk communication in clinical trials: A cognitive experiment and a survey

**DOI:** 10.1186/1472-6947-10-55

**Published:** 2010-09-27

**Authors:** Yin Bun Cheung, Hwee Lin Wee, Julian Thumboo, Cynthia Goh, Ricardo Pietrobon, Han Chong Toh, Yu Fen Yong, Say Beng Tan

**Affiliations:** 1 Center for Quantitative Medicine, Duke-NUS Graduate Medical School, College Road, 169857, Singapore; 2 Department of Biostatistics, Singapore Clinical Research Institute, Biopolis Way, 138669, Singapore; 3 Department of Pharmacy, National University of Singapore, Science Drive 4, 117543, Singapore; 4 Department of Rheumatology and Immunology, Singapore General Hospital, Outram Road, 169609, Singapore; 5 Department of Palliative Medicine, National Cancer Centre, Hospital Drive, 169610, Singapore; 6 Health Services Research Program, Duke-NUS Graduate Medical School, College Road, 169857, Singapore; 7 Department of Surgery, Duke University Medical Center, Durham, NC 27715, USA; 8 Department of Medical Oncology, National Cancer Centre, Hospital Drive, 169610, Singapore

## Abstract

**Background:**

A Royal Statistical Society Working Party recently recommended that "Greater use should be made of numerical, as opposed to verbal, descriptions of risk" in first-in-man clinical trials. This echoed the view of many clinicians and psychologists about risk communication. As the clinical trial industry expands rapidly across the globe, it is important to understand risk communication in Asian countries.

**Methods:**

We conducted a cognitive experiment about participation in a hypothetical clinical trial of a pain relief medication and a survey in cancer and arthritis patients in Singapore. In part 1 of the experiment, the patients received information about the risk of side effects in one of three formats (frequency, percentage and verbal descriptor) and in one of two sequences (from least to most severe and from most to least severe), and were asked about their willingness to participate. In part 2, the patients received information about the risk in all three formats, in the same sequence, and were again asked about their willingness to participate. A survey of preference for risk presentation methods and usage of verbal descriptors immediately followed.

**Results:**

Willingness to participate and the likelihood of changing one's decision were not affected by the risk presentation methods. Most patients indicated a preference for the frequency format, but patients with primary school or no formal education were indifferent. While the patients used the verbal descriptors "very common", "common" and "very rare" in ways similar to the European Commission's Guidelines, their usage of the descriptors "uncommon" and "rare" was substantially different from the EU's.

**Conclusion:**

In this sample of Asian cancer and arthritis patients, risk presentation format had no impact on willingness to participate in a clinical trial. However, there is a clear preference for the frequency format. The lay use of verbal descriptors was substantially different from the EU's.

## Background

Many studies on informed consent for clinical trials have demonstrated that people often have limited understanding about the trials they agreed to participate in [1]. An important consideration in accepting a medical product or participating in a trial is the risk of harm [2]. In 2006, a phase I trial at the Northwick Park Hospital, London, resulted in six volunteers suffering from life-threatening cytokine release syndrome [3]. In response to the Northwick Park Hospital incident, the Royal Statistical Society (RSS) commissioned a Working Party to investigate how to improve the conduct of first-in-man medical research. The RSS Working Party recommended [3], among other things, that "Greater use should be made of numerical, as opposed to verbal, descriptions of risk". This is not a new suggestion. In 2003, the Journal of the Royal Statistical Society, Series A [4], and the British Medical Journal [5] each published a special issue on risk communication, in which various statisticians and clinicians make similar suggestions. Some psychologists who specialised in decision making have also recommended the use of numbers, not words, for description of risk [6].

However, there are limited data and inconclusive evidence on whether risk presentation formats actually influence understanding and decision making in real or hypothetical clinical situations [7-13], although the evidence is strong that the use of frequency presentation facilitates accurate mathematical operations [7,14]. Studies that focused on comparison of relative versus absolute risk reduction or comparison of different graphical tools are important, but they are not directly relevant to the present theme and therefore not discussed here. A recently published systematic review focused on communicating cardiovascular risk information [12]. It concluded that "numerical presentation of risk as opposed to simple risk categories (e.g. high, moderate, low) appears to lead to more accurate risk perceptions", but "there is conflicting evidence regarding whether [different] numerical presentation formats may affect perceptions or emotions" [12]. Similarly, after a review of empirical findings and consultations with experts in risk communication, Lipkus [8] concluded that "few overall recommendations could be suggested" for the use of numeric, verbal and visual formats. A study of screening for Down's syndrome in pregnant women showed that 94 out of 97 (97%) and 102 out of 112 (91%) women who received test results in numerical probability and verbal description, respectively, correctly understood their test results, and that a normal approximation test gave a 1-sided p-value 0.04 for this difference in proportions [15]. However, a Fisher's exact test would give p-values 0.071 (1-sided) or 0.093 (2-sided) for this data. A recent online study of adult volunteers recruited from the general public found that the use of frequency presentation did not make a difference in decision to take a cholesterol lowering drug in a hypothetical scenario, but it was associated with higher satisfaction with information [16]. A small-scale study (n = 84) in Singapore on willingness to receive an influenza vaccine among healthcare workers and students showed only a small difference between those who received risk information in frequency versus percentage format [2]. All but one (Singapore) of the aforementioned or cited empirical studies were conducted in either Europe or North America; all but one (New Zealand) of the studies included in the systematic review of Waldron et al. [12] were conducted in either Europe or North America. There is a paucity of information about the impact of risk presentation formats in Asian culture.

A survey of patients who attended a university hospital in Japan found that more patients preferred the use of words only (41.4%) than numbers only (35.8%) in the communication of risk [17]. This is in sharp contrast with studies conducted in North America and Europe, which tended to suggest that respondents preferred to receive medical [18-21] and general probability information in numerical formats [22]. This highlights the importance of conducting research in a local setting. The Japanese survey [17] and a series of British survey [23,24] showed that there was a wide variation in the understanding and usage of verbal descriptors of drug side effects.

Another aspect of information presentation concerns the sequence of presenting side effects, e.g. from most to least severe or the other way round. The impact of this on patient decision remains largely unexplored [18]. It has been suggested that effectiveness of communication tends to increase when the communication is designed to respond to consumers' preferences [18,25]. It remains uncertain what the public in Asian countries prefer regarding risk presentation in medical care and clinical trials, and thus it is not clear how best to present information to potential clinical trial participants.

The expansion of the clinical trial industry around the globe, and especially in Asia, has been rapid in the last decade. There is not conclusive evidence about the impact of risk presentation formats and people's preference, especially in non-Caucasian populations. We therefore conducted an empirical study to examine risk communication and decision making in the context of clinical trial practice in Singapore. The study included a cognitive experiment and a post-experiment survey on preferences about risk communication. Three formats of risk presentation were considered, namely, frequency (e.g. 1 in 200), percentage (e.g. 0.5%) and verbal description (e.g. uncommon). The verbal description followed the European Union's guideline on drug labeling [26]. In this guideline, risk levels of ≤ 0.01%, > 0.01% to 0.1%, > 0.1% to 1%, > 1% to 10%, and > 10% were described as "very rare", "rare", "uncommon", "common" and "very common", respectively. The guideline was not based on patient inputs on the descriptors. It is not clear whether patients would interpret these risk levels as the EU intended [2,23]. The secondary purposes included comparing the impact of presenting information on risk of side effects in two different sequences, i.e. from most to least and from least to most severe, eliciting patient preference on risk communication, and exploring whether patients' usage of verbal descriptors of risk agrees with that of the EU.

## Methods

### Study design

Patients were recruited from an arthritis clinic of the Singapore General Hospital and the outpatient clinic of the National Cancer Center, Singapore. The study was approved by the Institutional Review Boards of the two institutions. Patients were recruited from the clinics while they were in the waiting area. A research assistant with a degree in psychology explained the study and obtained written informed consent. The first part of the study was a cognitive experiment, which used a factorial design to study the impact of the aforementioned three formats and two sequences (6 combinations) on willingness to participate and likelihood to change one's willingness after given additional information. Consented consecutive participants were presented with information in one of the 6 combinations in a pre-specified order, i.e. sequential allocation instead of true randomisation. A hypothetical situation about a clinical trial of a pain therapy was presented to each participant. It was made clear to the study participants that they were participating in a cognitive experiment and preference survey about risk communication and willingness to take part in a hypothetical clinical trial. The research assistant also stated that there were no right or wrong answers and that we were only interested in what the participant thought. Participants were given a Card 1 that showed information about side effects of a new medication for pain relief in one of the 6 ways of risk presentation, and then asked whether they would be willing to take part in this clinical trial. An example of card 1 presenting information in the frequency format and the least to most severe sequence is in Additional file 1. They were then presented with Card 2, with the same risk information presented in all three formats being studied (but in the same sequence in severity), and were asked for their decision again. The Card 2 that complements the Card 1 in Additional file 1 is also included in the Additional file. A change in decision would indicate a potential problem in the format the participants were given initially.

The cognitive experiment was followed by a short survey to assess the participants' preference for risk communication. The research assistant recapitulated that in the experiment just completed we had presented risk information in three different formats, and then asked the participant which of these formats s/he preferred most. In order to avoid the potential of an order effect [27,28], the sequence of the three formats (total six sequences) in the recapitulation were balanced across consecutive participants. To avoid unnecessary complexity, the six sequences in the preference section were linked to the six combinations of risk presentation in Card 1. For example, if Card 1 presented risk in percentage format from least to most severe side effects, the research assistant recapitulated that the experiment just completed had presented risk "in percentages (e.g. 0.2%), in descriptive terms (e.g. uncommon), and in frequencies (e.g. 1 out of 500)"; if Card 1 presented risk in percentage format from least to most severe side effects, the recapitulation was "in percentage (e.g. 0.2%), in frequencies (e.g. 1 out of 500) and descriptive terms (e.g. uncommon)". That is, the first format in the recapitulation was the format used in Card 1, whereas the second and third were determined by the severity sequence in Card 1. Furthermore, the participants were asked which one of the five EU descriptors ("very rare",..., "very common") best described the frequency of 1 out of 40, 1 out of 4,000, 1 out of 5, 1 out of 200 and 1 out of 20,000. The five questions were asked in this order. The reason of using this order instead of using an order with monotonic increase or decrease in risk presented was to avoid one answer being easily affected by the previous answer. These figures were chosen so that they covered each of the EU's descriptors from "very common" to "very rare".

### Statistical considerations

A previous cognitive experiment suggested that the percentages of people who change their treatment decision after given initial and then supplementary information range between 25% and 5% for different information sets [29]. A sample size of 80 per risk presentation format would give 90% power and 5% type I error rate in detecting this level of difference (based on a two-sample test of proportion 0.25 vs 0.05). A total sample size of 80×3 = 240 participants is needed. To explore whether there is a difference between participants with non-life-threatening versus life-threatening diseases, about one-third of the participants were recruited from the arthritis clinic and two-thirds from the cancer center. The number of arthritis and cancer patients offered about 90% power for detecting a 20% difference in willingness to participate. Only English speaking participants were recruited because the study involved the EU descriptors of risk, which are in English. In 2000, 71% of the Singaporean population was literate in English (http://www.singstat.gov.sg; accessed 28 Oct 2009), the lingua franca of Singapore. Fisher's exact test was used to compare distribution of categorical variables across groups of participants; McNemar test was used to compare change in willingness to participate; Binomial probability test was used to assess whether stated preferences were randomly distributed. All tests were conducted in Stata version 10.1 [30], using the "exact", "symmetry" and "bitest" functions.

## Results

### Participant characteristics

A total of 240 participants were recruited, of which 162 were cancer patients and 78 were arthritis patients. The average age was 52 years (SD = 13 and range from 21 to 87) and 58% were female (Table 1). Fifty-six participants had university degree or higher education level; 154 had secondary school or polytechnic diploma level. The distribution of the background characteristics was very similar across the three presentation formats (Table 1). The distribution was also very similar across the two sequences of presentation according to severity and across all six groups defined by the three formats and two sequences (details not shown in table).

**Table 1:** Patient characteristics (*N *= 240)

Variable	Category/statistics	All participants	Frequency group	Percentage group	Descriptors group	P*
Age (years)	Mean (SD)	51.5 (12.6)	52.3 (11.1)	51.6 (13.2)	50.6 (13.6)	0.694
Gender	Female	140 (58.3%)	35 (42.7%)	32 (40.0%)	33 (42.3%)	0.945
	Male	100 (41.7%)	47 (57.3%)	48 (60.0%)	45 (57.7%)	
Education	Primary or below	29 (12.1%)	12 (14.6%)	10 (12.5%)	7 (9.1%)	0.673
	Secondary or Diploma	154 (64.2%)	53 (64.6%)	48 (60.0%)	53 (68.8%)	
	Graduate or Postgraduate	56 (23.3%)	17 (20.7%)	22 (27.5%)	17 (22.1%)	
Marital status	Married	51 (21.3%)	18 (22.0%)	16 (20.0%)	17 (21.8%)	0.947
	Single	183 (76.3%)	61 (74.4%)	62 (77.5%)	60 (76.9%)	
	Divorce/Separated	6 (2.5%)	3 (3.7%)	2 (2.5%)	1 (1.3%)	
Ethnicity	Chinese	188 (78.3%)	64 (78.1%)	66 (82.5%)	58 (74.4%)	0.901
	Malay	21 (8.8%)	8 (9.8%)	5 (6.3%)	8 (10.3%)	
	Indian	16 (6.7%)	6 (7.3%)	4 (5.0%)	6 (7.7%)	
	Others	15 (6.3%)	4 (4.9%)	5 (6.3%)	6 (7.7%)	
Disease type	Arthritis	78 (32.5%)	28 (34.2%)	26 (32.5%)	24 (30.8%)	0.831
	Breast cancer	49 (20.4%)	17 (20.7%)	16 (20.0%)	16 (20.5%)	
	Colorectal cancer	25 (10.4%)	9 (11.0%)	7 (8.8%)	9 (11.5%)	
	Gynaecological cancer	13 (5.4%)	4 (4.9%)	7 (8.8%)	2 (2.6%)	
	Head and neck cancer	17 (7.1%)	5 (6.1%)	6 (7.5%)	6 (7.7%)	
	Lung cancer	18 (7.5%)	9 (11.0%)	5 (6.3%)	4 (5.1%)	
	Other cancers	40(16.7%)	10 (12.2%)	13 (16.3%)	17 (21.8%)	

* Test of difference between three formats: ANOVA for age; Fisher's exact test for categorical variables.

### Willingness and decision to participate

The number of participants who received Card 1 in frequency, percentage and verbal description formats were 82, 80 and 78, respectively (Table 2). After reading Card 1, 29, 26 and 28 participants in the frequency, percentage and verbal description group, respectively, indicated a willingness to participate in the trial. There was no significant difference between the three formats (P = 0.886). After reading Card 2, the proportion of participants indicating a willingness to participate changed to 51%, 44% and 46% in the frequency, percentage and verbal description groups, respectively. The increase within each group was statistically significant (each P < 0.05). There was no significant difference across the three groups in proportion of participants changing their mind (P = 0.529). There was no statistically significant pair-wise difference between the three groups in willingness to participate after Card1 and Card 2 or in the likelihood of changing decision (each P > 0.05). Among those who changed their mind, most changed from unwilling to willing to take part in the clinical trial. Test of equal proportion in each of these outcomes in arthritis versus cancer patients showed no significant difference (each P > 0.05; details not shown in table). Exploratory analyses repeating the above tabulations in participants with different levels of educational background showed similar results (details not shown in table).

**Table 2:** Willingness to participate, change of mind and whether format affects decision by risk presentation format

Response	Frequency (*n *= 82)	Percentage (*n *= 80)	Description (*n *= 78)	*P*-value
Willingness to participate after card1	29 (35.4%)	26 (32.5%)	28 (35.9%)	0.886
Willingness to participate after card2	42 (51.2%)	35 (43.8%)	36 (46.2%)	0.636
Change of mind	15 (18.3%)	11 (13.8%)	6 (20.5%)	0.529
Yes to No	1	1	4	
No to Yes	14	10	12	

Table 3 shows the willingness to participate after Card 1 and Card 2, and the proportion of participants who changed their mind after Card 2 for the two groups with side effects presented from increasing and decreasing severity. There results were similar across the two groups.

**Table 3:** Willingness to participate, change of mind and whether format affects decision by order of risk presentation

Response	Increasing severity (*n *= 120)	Decreasing severity (*n *= 120)	*P*-value
Willingness to participate after card1	44 (36.7%)	39 (32.5%)	0.587
Willingness to participate after card2	58 (48.3%)	55 (45.8%)	0.796
Change of mind	18 (15.0%)	24 (20.0%)	0.396
Yes to No	2	4	
No to Yes	16	20	

### Preference

**Figure 1:**
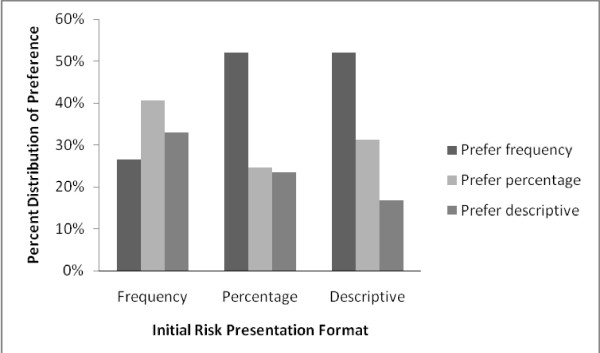
**Preference for risk presentation format, by actual risk presentation format in Card 1**.

**Figure 2:**
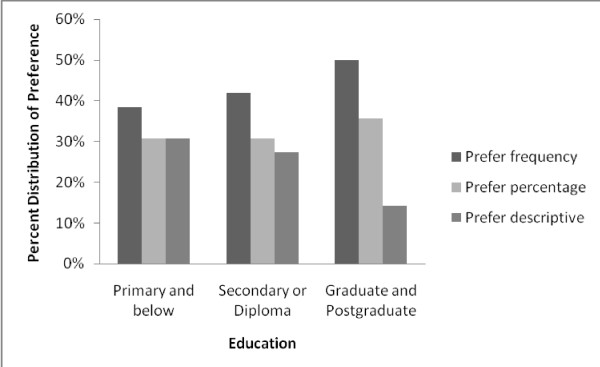
**Preference for risk presentation format, by educational background**.

When asked what their most preferred way of risk presentation format was (among the three used in the experiment), 43% indicated that they most preferred the frequency format, showing statistical significance against the null hypothesis of this being 1/3 (P = 0.001). Thirty two percent and 25% of the participants preferred the percentage and verbal description format respectively. Figure 1 shows the distribution of preferred formats by risk presentation formats in Card 1. Interestingly, within each group, participants tended to indicate a preference for a risk presentation format they did not initially received in Card 1. Figure 2 shows participant preference by education level. The higher the education level was, the stronger the preference for frequency format. Among participants with primary or below education background, the three formats were roughly equally preferred (P = 0.678).

### Verbal descriptions

**Figure 3:**
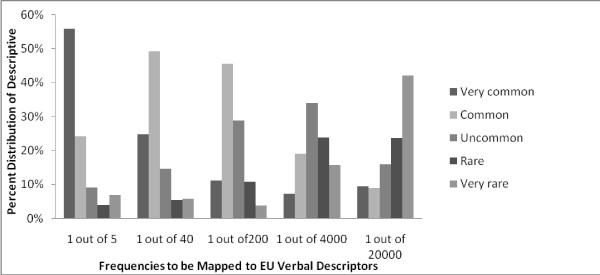
**Patients' verbal description of risk levels given in the frequency format**.

Figure 3 shows the EU descriptors that the participants assigned to the frequencies from 1 out of 5 to 1 out of 20,000. The x-axis shows the frequencies the participants were asked to describe using the EU descriptors. The five bars for each frequency show the percentage distribution of the five EU descriptors being chosen to describe the frequency shown on the x-axis. According to the EU guidelines, these frequencies of side effects should be described as "very rare", "rare", "uncommon", "common" and "very common", respectively. Agreement on the two most frequent (1/5 and 1/40) and the least frequent (1/20,000) were fairly strong, with almost half (56%, 49% and 42%, respectively) of the participants assigning the descriptors "very common", "common", and "very rare" to them. However, for the frequencies of 1 out of 200 and 1 out of 4,000, the agreement between the participants and the EU intended use of the descriptors were more limited. Only 29% of the participants rated 1 out of 200 as "uncommon" and 24% rated 1 out of 4,000 as "rare" (as EU intends). The modes were one grade above the EU guideline suggested: 45% of the participants considered 1 out of 200 common (which is "uncommon" as per EU) and 34% consider 1 out of 4,000 uncommon (which is "rare" as per EU)

## Discussion

The last decade has seen a significant rise in concerns about how risk is communicated [3-6]. These concerns involve the communication of risk to the general public, patients, users and potential users of screening or genetic tests. As medical research expands globally, concerns about understanding of trial information in studies conducted outside Western societies is also rising [31].

There have been strong advocates by clinicians, psychologists and statisticians for the use of numerical information in risk communication [4-6]. The RSS Working Party also suggested the use of numerical format for presenting medical risk in the recruitment of clinical trial participants [3]. However, there was no conclusive evidence as to whether doing this does have an influence on patients' understanding, decision making, and behaviour [7-13]. We thus conducted a cognitive experiment using a hypothetical clinical trial to shed light on this issue. We have studied willingness to participate, which had been shown to be predictive of actual participation in longitudinal studies [32-34]. Improving trial participation is of course but just one facet in the process of conducting a good clinical trial and there are other aspects which are just as important. We have also examined concordance in willingness to participate before and after more comprehensive presentation, which relate to understanding [29,35]. Furthermore, we explored patients' preference in risk presentation formats, usage of descriptive terms, and whether sequence of presenting more or less severe side effects would affect decisions.

We used a scenario of a pain relieving medication because pain is a near universal experience and therefore most participants could relate to this. The limitation of the study includes that willingness and decision to participate in clinical trials probably is influenced by the purpose and nature of treatment. So the present finding should not be over generalized to all other conditions and interventions. Another limitation is that allocation of Card 1 was sequential rather than randomised. In practice, we see no way how in this context recruitment could become selective according to the risk format in the next Card 1. Furthermore, the distribution of participant characteristics was very similar across groups (Table 1), strongly suggesting that the allocation was practically random. A third limitation is that we only used a limited number of endpoints to assess the impact of risk presentation formats. There are other potential endpoints such as satisfaction with information. But inclusion of more endpoints might compromise quality and execution of the study (e.g. by having too lengthy interview that might affect patients' attention/response) and we decided not to.

Our study found that patients who received risk information in the three formats (frequency, percentage and verbal description) and two sequences (least to most and most to least severe) showed no difference in willingness to participate in the trial. Furthermore, there was no difference in the likelihood of changing their mind after being given information in additional formats either. Our data suggests that the way of presenting information makes limited practical difference in willingness to participate in trials. The participants in all three groups had a tendency to change from unwilling to willing to participate after presented with Card 2. It might be that information presented in additional formats helped to reduce uncertainty or the feeling of being uncertain, which in turn contributed to re-evaluation of situation or decision making. However, the finding only applies to the present risk scenario. In other risk scenarios, there is no guarantee that additional presentation of information will lead to the same direction of change.

Nevertheless, the participants showed a clear preference for risk to be presented in the frequency format. This reinforces the recommendations from the clinical, psychology and statistics circles. Moreover, description in words had the smallest number of participants who preferred it. The finding is in contrast to that of the Japanese study [17]. It appears that there is substantial cultural diversity in this regard within the region.

A potential participant's willingness to participate in a trial is not purely determined by the risk of unwanted effects. There are other benefits and barriers involved in the decision making process. The apparent discrepancy between participants' preference for risk to be presented in the frequency format and the lack of effect on their willingness to participate in a trial suggests that, even though participants have their preference, there are multiple factors that affect willingness and decision to participate in clinical trials. Ways of presenting the risk of side effects do not dominate this decision making process.

To respect patients' preference, and based on the assumption that communication is more effective when it corresponds to consumers' preferences [18,25], one may consider the use of frequency format in presenting side effects in patient information sheets during clinical trial recruitment, if the clinical trial must chose to use one and only one format. However, our study shows that patients who received one format of risk information tended to indicate preference for some other format. They seemed not satisfied with only one way of communication. As such, we would recommend the use of multiple presentation formats to facilitate decision making, an example can be seen from our Card 2 in the Additional file.

The use of description of risk in words has been a debatable practice, as the understanding of vocabulary may vary from one culture to another and from one person to another. One way of presenting risk by words is to follow the EU's guideline on drug labeling. Our data suggest that the patients used these descriptors in ways somewhat different from the EU, although the agreement between the patients and the EU guideline for the more extreme frequencies was reasonably good in our opinion. Most of the patients describe the mid-range frequencies 1 out of 200 and 1 out of 4,000 as common and uncommon, respectively, while the EU descriptor for these were "uncommon" and "rare", respectively. The use of the EU descriptors only may give an impression of the side effects being less frequent than the patients think. Hence, the use of verbal descriptors only is not recommended.

The preference for risk of side effect being communicated in frequency format was clearly associated with higher level of educational background. Among those with primary school or no formal education background, there was practically no preference between the three choices: The distribution was consistent with random distribution (i.e. one-third for each of the three choices). In a traditional clinician-patient relationship, clinicians play a paternalistic role and patients play a trusting role [6]. It might be that these patients were more inclined to follow this tradition and therefore did not indicate a clear preference. It should be emphasized that this is neither a positive nor negative characteristic. However, it is imperative that clinicians and clinical trialists make every effort to effectively communicate with all patients and actively promote their understanding of the potential risk of treatments or trial participation. As secondary and tertiary education become more common, and in societies with higher educational coverage, the desire for the frequency format is likely to be stronger.

## Conclusions

In this sample of Asian cancer and arthritis patients, risk presentation format had no impact on willingness or change in willingness to participate in trials. However, there is a clear preference for the frequency format among patients with secondary or post-secondary education. The lay use of verbal descriptors was substantially different from the EU's.

## Competing interests

The authors declare that they have no competing interests.

## Authors' contributions

All authors except YFY participated in the design of the experiment and survey. YBC conceived the study, led the statistical analysis and co-wrote the first draft of the manuscript. YFY participated in the questionnaire design, performed part of the statistical analysis and co-wrote the first draft. All authors participated in critical revision of the manuscript and approved this submission.

## Pre-publication history

The pre-publication history for this paper can be accessed here:

http://www.biomedcentral.com/1472-6947/10/55/prepub

## Supplementary Material

Additional file 1 **Appendix 1**. Example of Card 1 using frequency format in the least to most severe sequence and the complementing Card 2 that uses all three formats in the same sequence. Click here for file
