# Supplementary material for: Risk communication in clinical trials: A cognitive experiment and a survey
Source: BMC Med Inform Decis Mak. 2010 Sep 27;10:55. doi: 10.1186/1472-6947-10-55 (PMC2949696; doi:10.1186/1472-6947-10-55)
Supplement: Additional file 1 — Appendix 1. Example of Card 1 using frequency format in the least to most severe sequence and the complementing Card 2 that uses all three formats in the same sequence. [file 1472-6947-10-55-S1.DOC]

**Appendix 1.** Example of Card 1 using frequency format in the least to most severe sequence and the complementing Card 2 that uses all three formats in the same sequence.

Card 1

You are invited to take part in a clinical trial to evaluate the effect of a new medication for pain relief. This medication is likely to relieve you of the worst pain you can imagine. This pain may be a headache, bone pain, back pain, nerve pain, etc.

This medication has a few side effects, namely:

1. **1 in 20** patients who take this medication may experience non-itchy skin rash that lasts for 3 days and will resolve by itself even if you continue the medication.
2. **1 in 200** patients who take this medication may experience stomach pain that goes off when you stop the medication.
3. **1 in 2,000** patients who take this medication may develop a stomach ulcer with bleeding.
4. **1 in 20,000** patients who take this medication may develop life-threatening hepatitis.

Card 2

You are invited to take part in a clinical trial to evaluate the effect of a new medication for pain relief. This medication is likely to relieve you of the worst pain you can imagine. This pain may be a headache, bone pain, back pain, nerve pain, etc.

This medication has a few side effects, namely:

(a) **1 in 20** patients, or **5%** of patients may experience non-itchy skin rash that lasts for 3 days and will resolve by itself even if you continue the medication. The chance that this will happen is **common**.

(b) **1 in 200** patients, or **0.5%** of patients may experience stomach pain that goes off when you stop the medication. The chance that this will happen is **uncommon**.

(c) **1 in 2,000** patients, or **0.05%** of patients may develop a stomach ulcer with bleeding. The chance that this will happen is **rare**.

(d) **1 in 20,000** patients, or **0.005%** of patients may develop life-threatening hepatitis. The chance that this will happen is **very rare**.
